# Nonbacterial Thrombotic Endocarditis Unmasking Concomitant Monoclonal Gammopathy of Undetermined Significance (MGUS) by Manifesting as Stroke

**DOI:** 10.7759/cureus.114409

**Published:** 2026-08-12

**Authors:** Varun H Rao, Muhammad Ali Akhtar, Mahpara Munir, Vaibhavkumar Falki, Adiraj Singh

**Affiliations:** 1 Internal Medicine, Hurley Medical Center/Michigan State University College of Human Medicine, Flint, USA

**Keywords:** igh/maf rearrangement, marantic endocarditis, mgus, monoclonal gammopathy of undetermined significance (mgus), nbte, nonbacterial thrombotic endocarditis (nbte), partial deletion of chromosome 13, plasma cell neoplasia

## Abstract

Nonbacterial thrombotic endocarditis (NBTE) is a rare condition characterized by sterile platelet-fibrin vegetations on cardiac valves in the absence of systemic infection. The pathogenesis of marantic endocarditis is driven by endothelial dysfunction and a systemic hypercoagulable state. In contrast to infective endocarditis, vegetations in NBTE lack significant inflammatory infiltrates and do not yield positive blood cultures. NBTE typically comes to clinical attention via systemic embolic events, with cerebrovascular accidents serving as a clinical hallmark and constituting over 50% of cases. While NBTE is commonly associated with mucin-producing adenocarcinomas of the lung, pancreas, and gastrointestinal tract, its occurrence secondary to hematological malignancies or precursor plasma cell dyscrasias like monoclonal gammopathy of undetermined significance (MGUS) is exceedingly rare, particularly in young individuals. We report the case of a previously healthy 35-year-old woman who presented with acute-onset blurred vision. Neuroimaging via magnetic resonance imaging (MRI) revealed an acute left occipital infarct along with multiple chronic infarcts, raising a strong suspicion of a recurrent embolic process. A transesophageal echocardiogram (TEE) demonstrated two vegetations on the aortic valve with moderate transvalvular regurgitation; in the context of persistent negative blood cultures, these findings supported the diagnosis of NBTE. The patient was managed with systemic anticoagulation. A comprehensive hypercoagulable and autoimmune workup revealed an elevated lambda free light chain level with a decreased kappa/lambda ratio. Subsequent bone marrow biopsy and cytogenetic analysis established a diagnosis of MGUS featuring high-risk genomic aberrations, specifically an immunoglobulin heavy chain/musculoaponeurotic fibrosarcoma (IGH/MAF) rearrangement and the loss of chromosome 13 in a polyploid (3n, 4n) background, findings consistent with plasma cell neoplasia. The prevalence of MGUS in individuals under the age of 40 is exceptionally low, estimated at less than 0.3%. This case underscores NBTE as a critical finding that can unmask underlying, atypical plasma cell neoplasms. It highlights the necessity of an exhaustive diagnostic evaluation for occult hematological disorders and high-risk cytogenetic features in young patients presenting with multi-territory embolic strokes.

## Introduction

Nonbacterial thrombotic endocarditis (NBTE) is a rare condition that is characterized by sterile platelet-fibrin vegetations on cardiac valves in the absence of infection. It is also known as marantic endocarditis. It is associated most commonly with advanced malignancy and autoimmune diseases. These vegetations can embolize, leading to ischemic strokes and infarcts in multiple vascular territories [1,2].

The pathogenesis of marantic endocarditis includes endothelial dysfunction and a hypercoagulable condition. In contrast to infective endocarditis, vegetations in marantic endocarditis lack significant inflammatory infiltrates and do not yield positive blood cultures [2]. Diagnosis is made clinically in the absence of infection and the presence of small sterile vegetations on cardiac valves visualized by transesophageal echocardiography [2,3].

Malignancy-associated NBTE is commonly seen with mucin-producing adenocarcinoma of the lungs, pancreas, and gastrointestinal tract [4]. NBTE has been occasionally reported in the setting of hematological malignancies such as monoclonal gammopathy of undetermined significance (MGUS) or plasma cell dyscrasias; however, such cases are less common [5].

We are presenting a case of a previously healthy 35-year-old woman who presented with ischemic stroke and was found to have NBTE. Further evaluation revealed an underlying MGUS, and genetic analysis showed loss of chromosome 13 in a polyploid background. MGUS is uncommon in individuals under the age of 40, and the estimated prevalence is below 0.3% [6]. This case highlights the importance of evaluating underlying malignancies and associated genetic linkage in young patients presenting with ischemic stroke and marantic endocarditis.

## Case presentation

A 35-year-old Caucasian woman presented with sudden-onset vision loss for three days, associated with dizziness and headache. She has no past medical or surgical history and does not take any medications or supplements. She is nulliparous with a regular menstrual cycle. She lives alone, works as a freelance photographer and in the cafeteria of a high school, and has been sexually active with one partner for the past six months. She denies recreational drug or tobacco use. Review of systems was notable for intermittent palpitations over the past few years. There is no significant family history of hematological malignancy or autoimmune diseases. 

**Figure 1:**
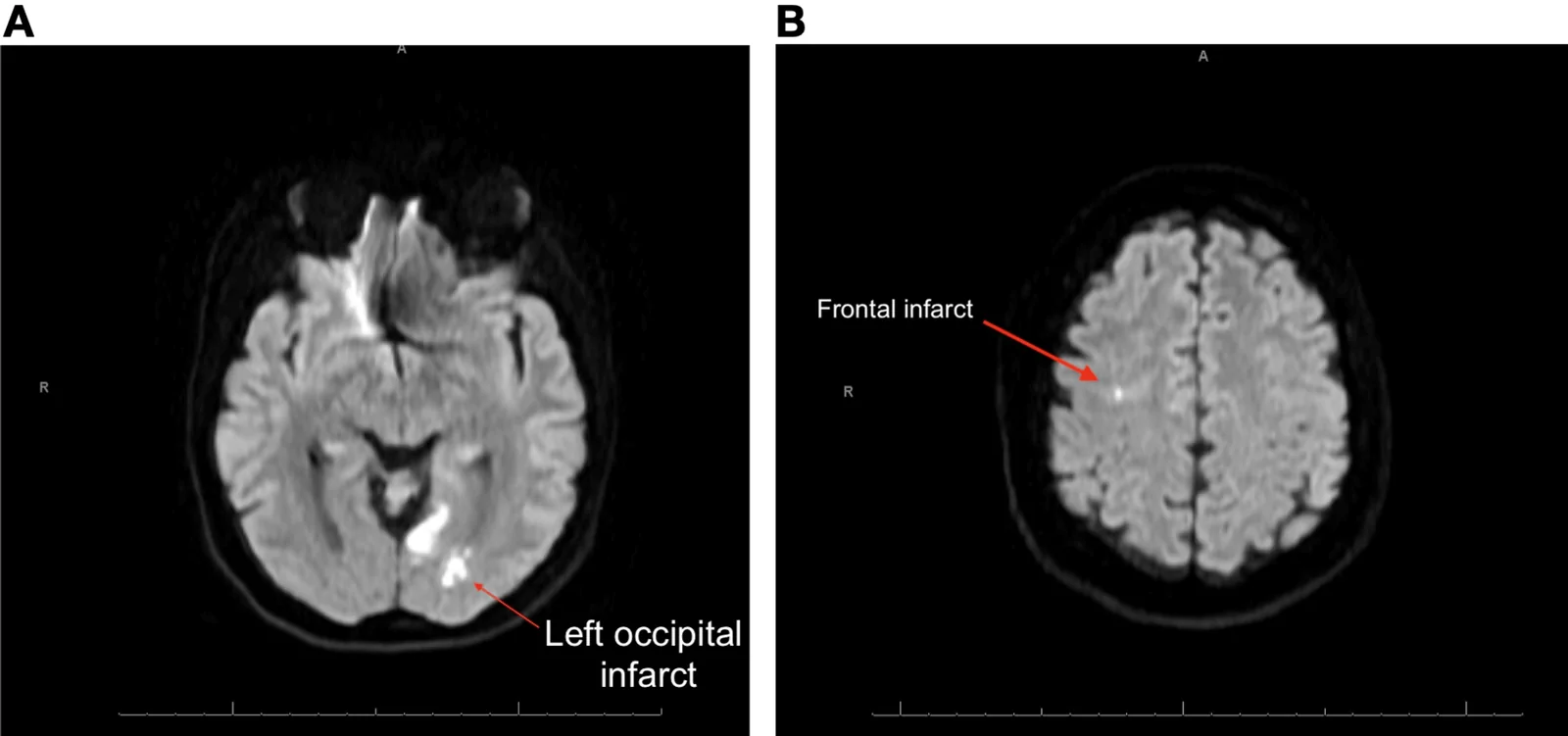
Brain magnetic resonance imaging MRI demonstrating multiterritory embolic infarctions MRI: magnetic resonance imaging MRI of the brain without contrast. (A) Axial diffusion-weighted imaging (DWI) demonstrating a focal area of acute hyperintense signal within the left occipital lobe corresponding to an acute ischemic infarct. (B) Separate axial sequence slice demonstrating a small acute ischemic focus bordering a chronic, remote infarct within the right frontal lobe. The presence of multiterritory cortical lesions of varying chronologies strongly indicates a recurrent central embolic etiology

**Figure 2:**
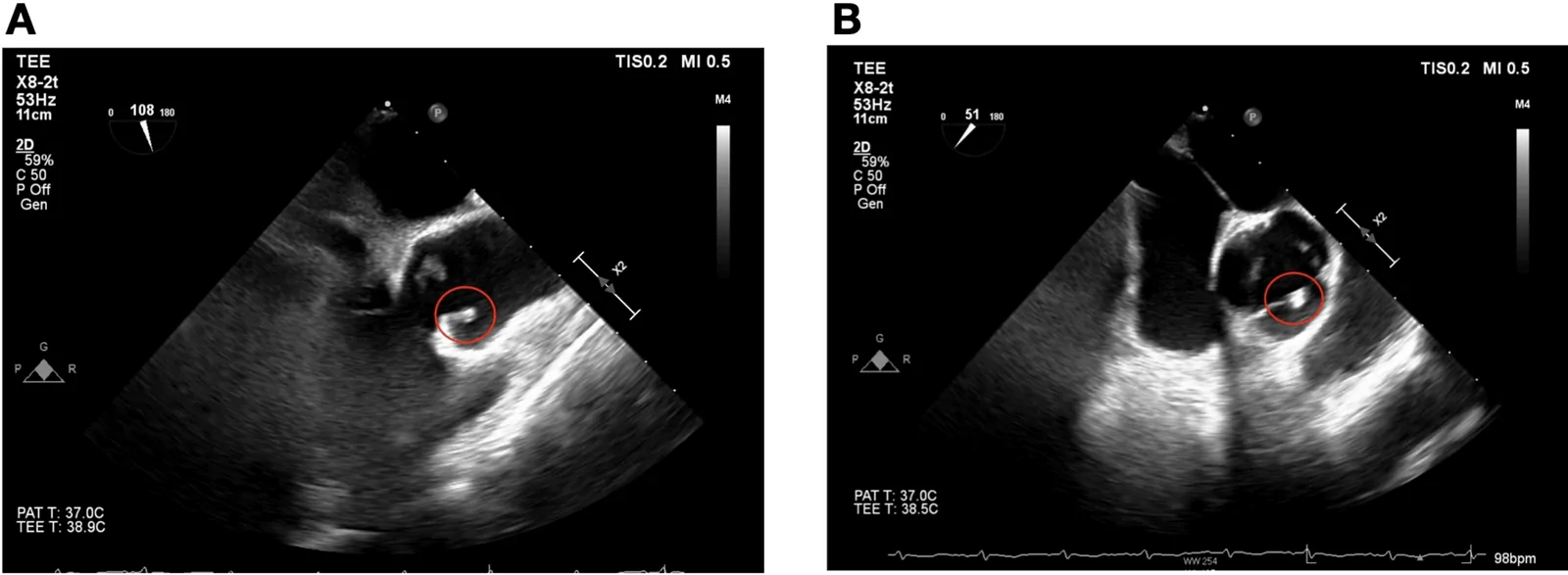
Transesophageal echocardiogram showing aortic valve vegetation TEE: transesophageal echocardiogram; NBTE: nonbacterial thrombotic endocarditis; ME AV LAX: mid-esophageal aortic valve long-axis; ME AV SAX: mid-esophageal aortic valve short-axis TEE demonstrating NBTE. (A) ME AV LAX view and (B) ME AV SAX view visualize a small, discrete lesion present on the aortic side of the right coronary cusp consistent with a sterile platelet-fibrin vegetation

**Figure 3:**
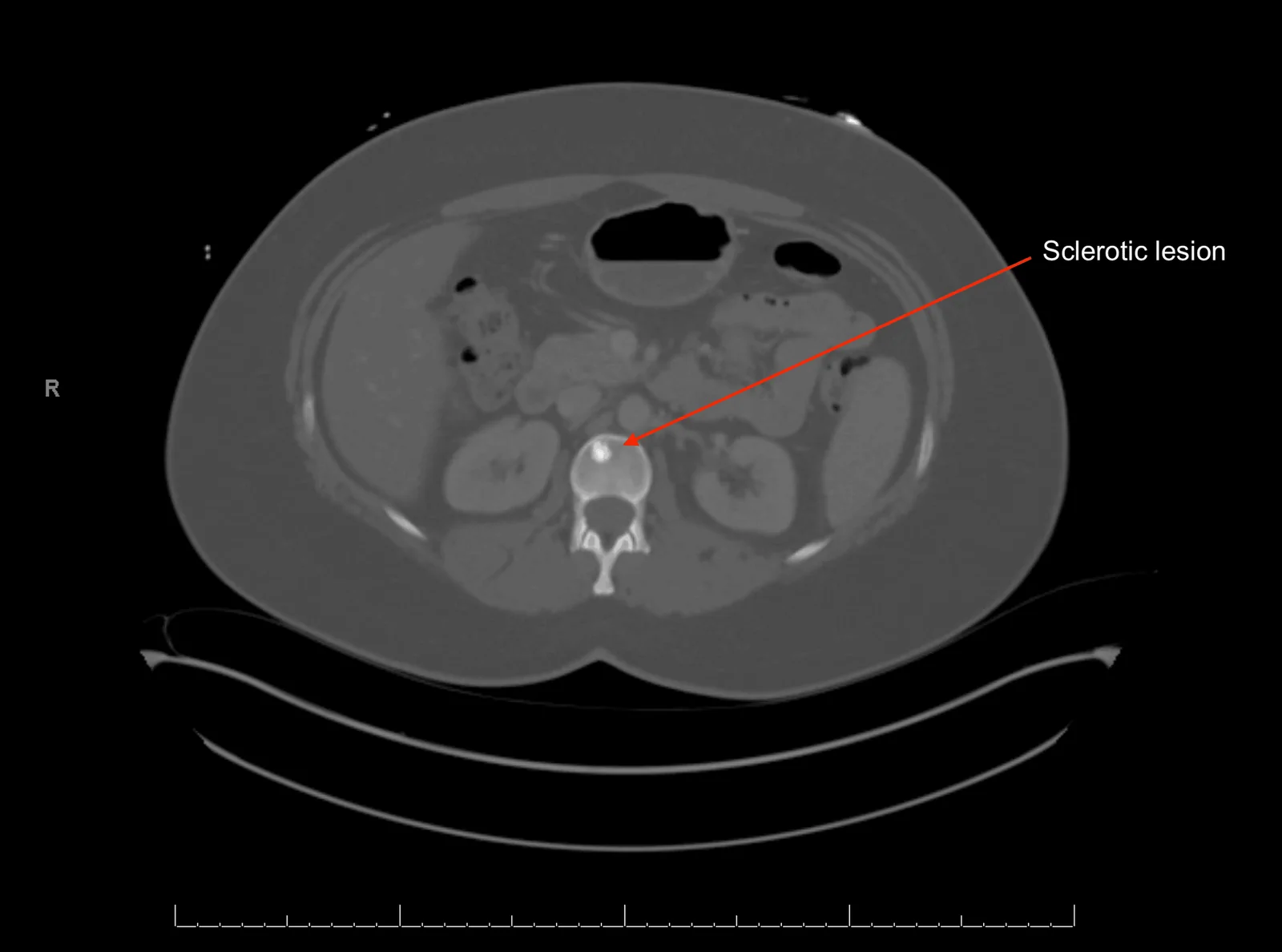
Computed tomography (CT) chest demonstrating a small focal sclerotic focus seen at L2 vertebral body measuring up to 1.1 cm in size

On examination, the only significant finding was right homonymous hemianopsia. Computed tomography (CT) of the head confirmed an acute/subacute ischemic infarction in the left occipital lobe. Magnetic resonance imaging (MRI) of the brain revealed acute left occipital infarcts, a small acute ischemic focus within a chronic right frontal infarct, and additional chronic infarcts, suggesting an embolic etiology (Figure 1). Transthoracic echocardiography did not reveal any vegetations or structural heart disease; the bubble study was negative. Transesophageal echocardiogram (TEE) demonstrated two small-to-moderate lesions on the right and noncoronary cusps of the aortic valve (Figure 2). Two sets of blood cultures showed no growth after five days. Serologies for *Neisseria gonorrhoeae*, *Chlamydia trachomatis*, and *Bartonella henselae* were negative. These findings supported the diagnosis of NBTE. The patient was started on anticoagulation with heparin, transitioned to subcutaneous enoxaparin on discharge along with atorvastatin and antiplatelet therapy with aspirin. Further hypercoagulable and autoimmune workup revealed elevated lambda free light chains with a decreased kappa/lambda ratio; a comprehensive compilation of these baseline laboratory evaluation metrics and normal reference intervals is detailed in Table 1. Hematology/oncology was consulted. A chest CT demonstrated a small focal sclerotic focus seen at the second lumbar (L2) vertebral body measuring up to 1.1 cm in size (Figure 3). Chromosome analysis and fluorescence in situ hybridization (FISH) testing of bone marrow biopsy showed immunoglobulin heavy chain/musculoaponeurotic fibrosarcoma (IGH/MAF) rearrangement and loss of chromosome 13 in a polyploid(3n, 4n) background, findings consistent with plasma cell neoplasia.

**Table 1:** Comprehensive laboratory and diagnostic workup

Test	Result	Reference range
Complete blood count (CBC) & electrolytes	Within normal limits	Normal
Electrocardiogram (EKG)	Sinus rhythm	Normal
Erythrocyte sedimentation rate (ESR)	28 mm/hr	0-20 mm/hr
C3 complement	181 mg/dL	90-180 mg/dL
Homocysteine	13.5 µmol/L	5.0-13.0 µmol/L
Serum protein electrophoresis-total protein	8.2 g/dL (monoclonal spike detected)	6.4-8.2 g/dL
Albumin (%)	46.5%	52.9-66.9%
Alpha-2-globulin	1.1 g/dL	0.5-0.9 g/dL
Gamma globulin (%)	26.6%	8.8-19.2%
Gamma globulin (g/dL)	2.2 g/dL	0.6-1.4 g/dL
IgG	2690 mg/dL	700-1600 mg/dL
Free light chain (lambda)	5.05 mg/dL	0.57-2.63 mg/dL
Kappa/lambda ratio	0.25	0.26-1.65
Thyroid-peroxidase antibodies	Positive	Negative
Human papillomavirus (HPV) test (2016; 9 years prior)	HPV-18 positive	Negative
Pap smear (follow-up)	Atypical squamous cells of undetermined significance (ASC-US)	Normal cytology
HPV test (follow-up)	Negative	Negative

## Discussion

NBTE is a rarely encountered manifestation of a preexisting hypercoagulable state characterized by sterile yet friable vegetations on cardiac valves with high susceptibility for systemic embolization [7]. NBTE typically comes to clinical attention in the form of embolic events, with cerebrovascular accidents being a hallmark, constituting over 50% of the cases [8]. We report a case of an apparently healthy, young female who presented with acute-onset blurred vision. Neuroimaging revealed an acute occipital infarct along with multiple chronic infarcts, raising strong suspicion of an underlying embolic process. Given this pattern, identifying the source was deemed necessary. In young patients presenting with cryptogenic, multiterritory embolic strokes, NBTE must be strongly considered in the differential diagnosis, particularly when standard workups for common cardiac sources (such as infective endocarditis, structural valvular disease, or patent foramen ovale) prove negative.

Additionally, our patient also complained of intermittent palpitations, which further prompted us to evaluate for potential cardiac etiologies. TEE demonstrating two vegetations on the aortic valve with moderate transvalvular regurgitation in the context of negative blood cultures supported the diagnosis of NBTE. Valvular involvement, although less frequent, is well-recognized in NBTE, with the mitral valve affected in 61% of the cases and the aortic valve involved in 23% of the cases [9]. This case emphasizes that meticulous and thorough evaluation is essential for physicians to identify the underlying cause in such patients.

In our case, initial presentation as a stroke, the presence of remote infarcts on imaging, and the young age of 35 followed by the diagnosis of NBTE sparked tremendous clinical curiosity. Literature consistently shows that NBTE is rarely an isolated entity and often serves as a clue warranting further investigation for an underlying autoimmune condition, hypercoagulable state, or occult malignancy. Among associated malignancies, lung cancer is the most prevalent, followed by pancreatic, gynecological, and gastrointestinal cancers [10]. A retrospective cohort study conducted across multiple regions in the USA from 1992 to 2020 identified a rare association between NBTE and hematological malignancies, constituting only 8.7% of the cases [11]. Extensive hypercoagulable and autoimmune workup was pursued. Therefore, upon identifying NBTE, an exhaustive investigation for an underlying occult malignancy or systemic hypercoagulable state is mandatory. 

The scenario took a turn when the results of bone biopsy revealed the diagnosis of MGUS. MGUS is a benign precursor condition; however, it carries potential for malignant transformation. Given its relevance to our case, we are adding a literature review as a key reference, which summarizes case reports and case series of NBTE associated with hematological disorders, yet none linked with MGUS [12]. The identification of MGUS in this context requires careful risk stratification and long-term surveillance. The overall risk of progression from MGUS to multiple myeloma or related disorders is approximately 1% per year [13]. However, the presence of unfavorable cytogenetic features, specifically the IGH/MAF rearrangement and loss of chromosome 13 identified in our patient, along with an abnormal kappa/lambda ratio and elevated lambda free light chains, places this patient in a higher risk category. Current risk stratification models suggest that patients with multiple adverse features may have a 20-year progression risk exceeding 50% [13,14]. Genomic features, particularly high-risk cytogenetic abnormalities, are increasingly recognized as important determinants of malignant transformation risk, and the polyploid background with chromosome 13 loss warrants heightened clinical vigilance [15]. 

This case is exceptionally unique as the diagnosis of MGUS was made in a 35-year-old patient, an age group where the prevalence is less than 0.3% [16]. Her plasma cell dyscrasia was unmasked by a cryptogenic event that led to the discovery of NBTE. Furthermore, her bone marrow evaluation revealed high-risk cytogenetic abnormalities, specifically an IGH/MAF translocation and chromosome 13 deletion, which are atypical for standard MGUS. This case highlights the complex relationship between plasma cell dyscrasias and thrombotic complications.

While MGUS is generally considered clinically benign, emerging evidence demonstrates that patients with MGUS have an increased risk of venous thromboembolism compared to matched controls [17]. The mechanisms underlying this hypercoagulable state are multifactorial and driven by cytokines and circulating paraproteins [18,19]. Monoclonal immunoglobulins increase plasma hyperviscosity and directly interfere with normal fibrin structure and polymerization [19]. Furthermore, clonal plasma cells drive the release of pro-inflammatory cytokines, which lead to endothelial injury and upregulate vascular adhesion molecules along the endocardium, causing acquired activated protein C (APC) resistance and tissue factor expression, ultimately driving thrombin generation [18]. Finally, the interaction of abnormal immunoglobulins, plasma cofactors, and damaged valvular endothelium promotes platelet activation and cellular adhesion, culminating in the formation of sterile, platelet-fibrin vegetations [19]. This development of NBTE in the context of MGUS represents a form of monoclonal gammopathy of thrombotic significance and may indicate more aggressive disease biology requiring closer monitoring.

Treatment strategies for NBTE focus on two main objectives, including mitigation of recurrence by treating predisposing conditions and preventing systemic embolization; however, in selected cases, surgical intervention might be necessary. Systemic anticoagulation options, including low-molecular-weight heparin (LMWH), vitamin K antagonist (warfarin), and recently direct oral anticoagulants (DOACs) (apixaban and rivaroxaban), are being used. Recent evidence suggests that DOACs may be as effective as traditional heparin-based therapy for preventing recurrent embolic events, while potentially reducing hospital length of stay [20]. However, a retrospective cohort study at the Mayo Clinic evaluating treatment outcomes across LMWH, warfarin, DOACs, and antiplatelet therapies in cancer-associated NBTE yielded no statistically significant differences regarding embolic recurrence or major bleeding risks among the groups [11]. The optimal duration and intensity of anticoagulation in MGUS-associated NBTE remains undefined. Simultaneously, ongoing hematologic surveillance is essential, with current guidelines recommending monitoring intervals tailored to risk stratification, typically ranging from every six months for high-risk patients to every 2-3 years for stable low-risk disease [14,15].

## Conclusions

This case demonstrates a rare clinical presentation of NBTE in a young, previously healthy patient without an obvious underlying malignancy, ultimately leading to the diagnosis of MGUS with high-risk cytogenetic features and concurrent autoimmune thyroiditis. First, NBTE should be considered in the differential diagnosis of cryptogenic stroke in young patients, particularly when imaging reveals multiple embolic events across different vascular territories. While NBTE is classically associated with advanced malignancy, this case illustrates that it can be the initial manifestation of occult hematological disorders, including premalignant conditions like MGUS. Second, the identification of MGUS in this context requires careful risk stratification and long-term surveillance. 

In summary, this case exemplifies how NBTE can serve as a sentinel event unmasking occult hematological and autoimmune conditions in otherwise healthy young individuals. It emphasizes the need for thorough investigation of embolic stroke in young patients, appropriate risk stratification of plasma cell disorders based on both clinical and genomic features, recognition of MGUS as a potential cause of hypercoagulability, and implementation of individualized monitoring and treatment strategies. Our case is unique given the unusual age of presentation of MGUS. Secondly, the role of chromosome 13 deletion is unclear. The proportion of plasma cells with chromosomal 13q deletions is lowest in patients with MGUS, and highest in patients with multiple myeloma. Our patient had a loss of chromosome 13, and she had MGUS and not multiple myeloma. 
